# Crystal structures and conformations of two Diels–Alder adduct derivatives: 1,8-bis­(thio­phen-2-yl)-14-oxa­tetra­cyclo­[6.5.1.0^2,7^.0^9,13^]tetra­deca-2(7),3,5-trien-10-one and 1,8-diphenyl-14-oxa­tetra­cyclo[6.5.1.0^2,7^.0^9,13^] tetra­deca-2,4,6-trien-10-one

**DOI:** 10.1107/S2056989015001073

**Published:** 2015-01-24

**Authors:** S. Gopinath, P. Narayanan, K. Sethusankar, Meganathan Nandakumar, Arasambattu K. Mohanakrishnan

**Affiliations:** a Department of Physics, RKM Vivekananda College (Autonomous), Chennai 600 004, India; b Department of Organic Chemistry, University of Madras, Guindy Campus, Chennai 600 025, India

**Keywords:** crystal structure, Diels–Alder adduct derivative, thio­phene, conformation, hydrogen bonding, C—H⋯π inter­actions, non-merohedral twin

## Abstract

The title compounds, C_21_H_16_O_2_S_2_ (I) and C_25_H_20_O_2_ (II), are products of a tandem ‘pincer’ Diels–Alder reaction consisting of [2 + 2] cyclo­additions between benzo[*c*]furan and cyclo­penta­none. Each comprises a fused tetra­cyclic ring system containing two five-membered rings (in *envelope* conformations with the O atom as the flap) and six-membered rings (in *boat* conformations). In addition, two thio­phene rings in (I) and two phenyl rings in (II) are attached to the tetra­cyclic ring system. The cyclo­penta­none ring adopts a *twisted* conformation in (I) and an *envelope* conformation in (II). In (I), the thio­phene rings are positionally disordered over two sets of sites, with occupancy ratios of 0.901 (2):0.099 (2) and 0.666 (2):0.334 (2). In (II), the oxygen atom of the cyclo­penta­none ring is rotationally disordered over two sites with an occupancy ratio of 0.579 (4):0.421 (4). The mol­ecular structure of (I) is stabilized by an intra­molecular C—H⋯O hydrogen bond, which generates an *S*(7) ring motif. In the crystal, the mol­ecules are linked *via* weak C—H⋯O hydrogen bonds, which generate *R* ^2^_2_(16) ring motifs in (I) and *C*(8) chains in (II). In both structures, the crystal packing also features C—H⋯π inter­actions. The crystal studied of compound (I) was twinned by non-merohedry. The twin component is related by the twin law [−1 0 0 −0.101 1 −0.484 0 0 −1] operated by a twofold rotation axis parallel to the *b* axis. The structure of (I) was refined with a twin scale factor of 0.275 (2).

## Abstract 2

The title compounds are the product of a tandem ‘pincer’ Diels–Alder reaction consisting of [2 + 2] cyclo­additions between benzo[*c*]furan and cyclo­penta­none. The mol­ecules are linked *via* weak C—H⋯O inter­molecular hydrogen bonds, which generate 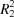
(16) ring motifs in compound (I) and *C*(8) chains in compound (II). In both structures, the crystal packing also features C—H⋯π inter­actions.

## Chemical Context   

The tandem ‘pincer’ Diels–Alder reaction, consisting of two consecutive [2 + 2] cyclo­additions between two dienes and an acetyl­enic bis-dienophile, when furan derivatives are used as the diene components (Lautens & Fillion, 1997 ▸). The Diels–Alder reaction is among the most powerful C—C-bond-forming processes and one of the most widely used and studied transformations in organic chemistry (Denmark & Thorarensen, 1996 ▸). Thio­phene derivatives are very important heterocyclic compounds, which possess anti­tubercular (Parai *et al.*, 2008 ▸), anti-depressant (Wardakhan *et al.*, 2008 ▸), anti-inflammatory (Kumar *et al.*, 2004 ▸), anti-HIV (Bonini *et al.*, 2005 ▸) and anti-breast cancer activities (Brault *et al.*, 2005 ▸). Against this background, the conformational studies and X-ray structure determination of the title compounds have been carried out and the results are presented here.

## Structural Commentary   

**Figure 1:**
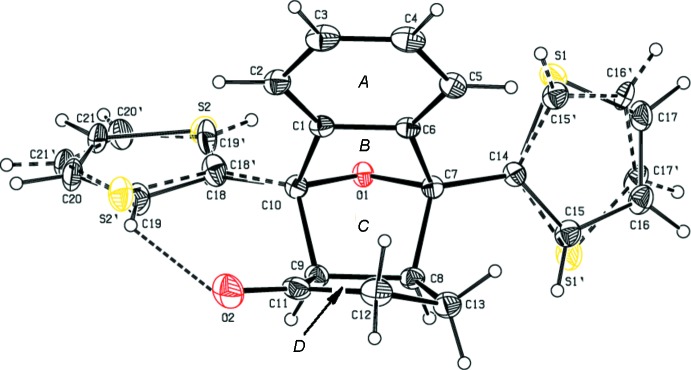
The mol­ecular structure of compound (I) is stabilized by a C19—H19⋯O2 intra­molecular inter­action (dashed line), which generates an *S(7)* ring motif. Displacement ellipsoids are drawn at the 30% probability level.

The mol­ecular structures of (I) and (II) are shown in Figs. 1 ▸ and 2 ▸, respectively, along with the atomic as well as ring-labelling schemes. Both compounds exhibit disorder, *viz*., in the thio­phene rings of (I) and the oxygen atom of the cyclo­penta­none ring in (II). Further details are given in the *Refinement* section.
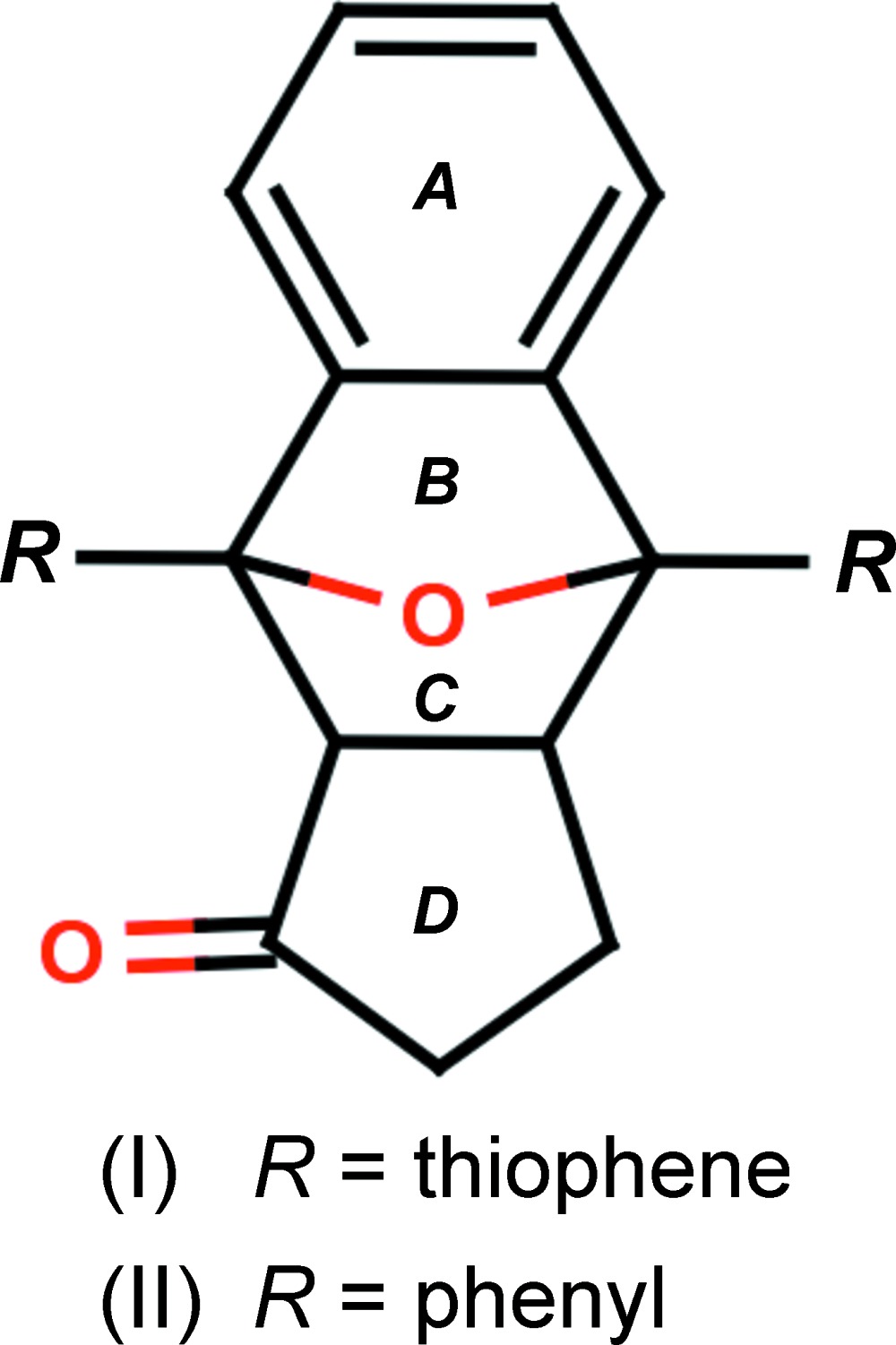


Rings *B* and *C* adopt an *envelope* conformation in both compounds with atom O1 as the flap. In compound (I), the puckering parameters (Cremer & Pople, 1975 ▸) and smallest displacements parameters (Nardelli, 1983 ▸) are *q*_2_ = 0.5246 (15) Å, φ = 358.41 (18)°, ΔC_s_ = 2.45 (14) for *B* and *q*_2_ = 0.5819 (15) Å, φ = 185.54 (17)°, ΔC_s_ = 6.26 (14) for *C*. In compound (II) they are *q*_2_ = 0.5093 (16) Å, φ = 360.0 (2)°, ΔC_s_ = 0.01 (15) for *B* and *q*_2_ = 0.5585 (15) Å, φ = 179.53 (18)°, ΔC_s_ = 0.44 (14) for *C*. Cyclo­penta­none ring *D* adopts a *twisted* conformation on C12–C13 in (I) with puckering and smallest displacement parameters of *q*_2_ = 0.184 (2) Å, φ = 133.7 (6)°, ΔC_2_ = 3.65 (19) whereas in (II), this ring adopts an *envelope* conformation on C12 with puckering and smallest displacement parameters of *q*_2_ = 0.265 (2) Å, φ = 290.1 (5)°, ΔC_2_ = 1.1 (2).

In both compounds, the cyclo­hexane ring embracing rings *B* and *C* (C1/C6–C10) adopts a *boat* conformation with puckering amplitude and smallest displacement parameters of *q* = 0.9648 (17) Å, θ = 88.53 (10), φ = 296.96 (10)° and ΔC_s_ = 6.45 (15) in (I) and *q* = 1.0000 (18) Å, θ = 90.16 (10), φ = 300.17 (10)° and ΔC_s_ = 0.72 (15) in (II).

Rings *A* and *D* in (I) form dihedral angles of 57.02 (14) and 82.70 (14)°, respectively, with the S1/C14–C17 thio­phene ring (major occupancy component) and 62.9 (3) and 20.7 (3)°, respectively, with the major component of the S2/C18–C21 thio­phene ring. In (II), rings *A* and *D* subtend angles of 65.03 (9) and 71.65 (11)°, respectively with phenyl ring C14–C19, and 65.88 (10) and 72.51 (12)°, respectively, with phenyl ring C20–C25. The dihedral angle between the thio­phene rings in (I) is 70.3 (3)° and that between the phenyl rings in (II) is 6.93 (10)°. In both compounds, rings *B* and *C* are almost perpendicular to each other [dihedral angles of 83.61 (10) and 82.26 (10)°, respectively].

In compound (I), an intra­molecular hydrogen bond, C19—H19⋯O2, occurs, which generates an *S*(7) ring motif (Table 1 ▸).

## Supra­molecular features   

**Figure 3:**
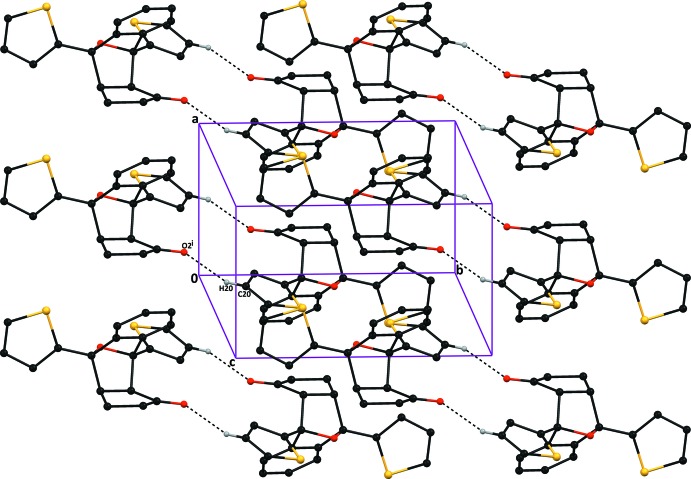
The crystal packing of compound (I), viewed down the *a* axis, showing the C20—H20⋯O2^i^ inter­molecular hydrogen bond (dashed lines), which results in 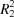
(16) ring motifs. Hydrogen atoms not involved in this hydrogen bond are excluded for clarity. [Symmetry code: (i) 1 − *x*, −*y*, 1 − *z*.]

In both structures, the crystal packing features C—H⋯O and C—H⋯π inter­actions (Tables 1 ▸ and 2 ▸). In compound (I), the C20—H20⋯O2(−*x* + 1, −*y*, −*z* + 1) hydrogen bond generates an 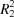
(16) graph-set ring motifs around an inversion centre (Bernstein *et al.*, 1995 ▸) while in compound (II), the weak C5—H5⋯O2(*x* − 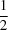, −*y* + 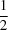, *z* − 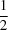) hydrogen bond generates *C(8)* chains running parallel to the *c* axis. The resulting packing in (I) and (II) is shown in Figs. 3 ▸ and 4 ▸, respectively. The structures of both compounds also feature C—H⋯π inter­actions (Tables 1 ▸ and 2 ▸).

## Synthesis and crystallization   

The title compounds were prepared in a similar manner using a solution of 1,3-bis­thio­phen-2-yl-2-benzo­furan (0.30 g, 1.00 mmol) in dry 1,2-DCE (20 mL) for compound (I) and a solution of 1,3-diphenyl-2-benzo­furan (0.30 g, 1.011 mmol) in dry DCE (20 mL) for compound (II). 2-Cyclo­pentenone was added in both cases [0.104 g, 1.2 mmol for (I), 0.11 g, 1.33 mmol for (II)] and refluxed until the disappearance of the fluorescent colour of 1,3-bis­thio­phen-2-yl-2-benzo­furan or 1,3-diphenyl-2-benzo­furan (10 h). Removal of the solvents was followed by column chromatographic purification (silica gel; 10% ethyl acetate in hexa­ne), affording the adduct as a colourless solid for both (I) (yield = 0.23 g, 61%) and (II) (yield = 0.27 g, 68%). Single crystals suitable for X-ray diffraction were prepared by slow evaporation of a solution of compound (I) or (II) in ethyl acetate at room temperature.

## Refinement   

Crystal data, data collection and structure refinement details are summarized in Table 3 ▸. Compound (I) initially refined to a high *R* index of 0.103 (2) and the difference Fourier map showed relatively larger peaks [Δρ_max_ = 0.97 (2) e Å^−3^]. A preliminary check with *TWINLAW* (Bolte, 2004 ▸) showed that the crystal had twofold twinning by non-merohedry about [001] with a twin matrix of [−1 00 −0.101 1 −0.484 0 0 −1]. The twin law operated from the *F*_o_–*F*_c_ table was used to a generate an HKLF5 format file (Bolte, 2004 ▸) suitable for twin refinement in *SHELXL97* (Sheldrick, 2015 ▸). The twinning was a twofold rotation axis parallel to the *b* axis with a refined twin scale factor of 0.275 (2). The structure was refined to an improved *R* index of 0.064 (2) with an essentially flatter difference Fourier map [Δρ_max_ = 0.38 (2) e Å^−3^].

The positions of the hydrogen atoms were localized from difference electron density maps and further idealized and treated as riding atoms, with *d*(C—H) = 0.93, 0.97 and 0.98 Å for aryl, methyl­ene and methine H atoms, respectively, and *U*_iso_(H) = 1.2*U*_eq_(C). In compound (I), the thio­phene rings C11–C14/S1 and C15–C18/S2 are disordered over two sets of sites with occupancy ratios of 0.901 (2):0.099 (2) and 0.666 (2):0.334 (2), respectively. Geometrical (FLAT) restraints were applied to both the major and minor components of the thio­phene ring atoms to keep the rings planar. Ellipsoid displacement (SIMU and DELU) restraints were also applied to the disordered rings. In compound (II), the oxygen atom O2 is disordered with an occupancy ratio of 0.579 (4):0.421 (4). In both compounds, bond lengths for both major and minor components were restrained to standard values using the command DFIX (s.u. 0.01 Å) in *SHELXL97* (Sheldrick, 2015 ▸).

## Supplementary Material

Crystal structure: contains datablock(s) I, II, global. DOI: 10.1107/S2056989015001073/bg2544sup1.cif

Structure factors: contains datablock(s) I. DOI: 10.1107/S2056989015001073/bg2544Isup2.hkl

Structure factors: contains datablock(s) II. DOI: 10.1107/S2056989015001073/bg2544IIsup3.hkl

Supporting information file. DOI: 10.1107/S2056989015001073/bg2544Isup4.cml

Supporting information file. DOI: 10.1107/S2056989015001073/bg2544IIsup5.cml

CCDC references: 997381, 997382

Additional supporting information:  crystallographic information; 3D view; checkCIF report

## Figures and Tables

**Figure 2:**
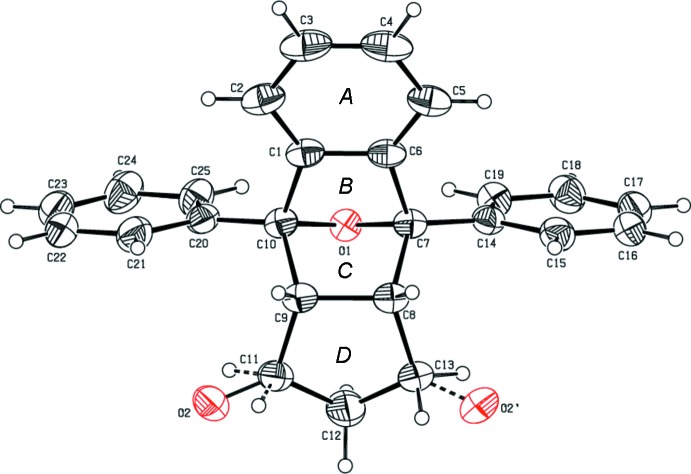
The mol­ecular structure of compound (II) with the atom-numbering scheme. Displacement ellipsoids are drawn at the 30% probability level.

**Figure 4:**
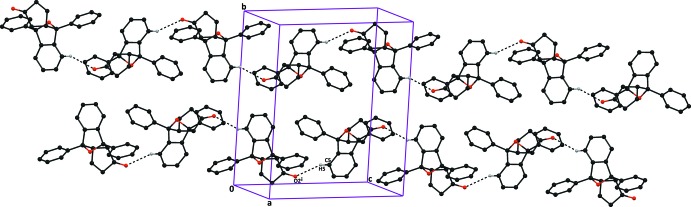
The crystal packing of compound (II), viewed down the *b* axis, showing the C5—H5⋯O2^i^ hydrogen bonds (dashed lines), which result in the formation of *C*(8) chains. Hydrogen atoms not involved in this hydrogen bond are excluded for clarity. [Symmetry code: (i) *x* − 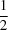, −*y* + 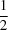, *z* − 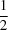.]

**Table 1:** Hydrogen-bond geometry (Å, °) for (I) *Cg*1 and *Cg*2 are the centroids of the S1,C14–C17 and S2/C18–C21 rings, respectively.

*D*—H⋯*A*	*D*—H	H⋯*A*	*D*⋯*A*	*D*—H⋯*A*
C19—H19⋯O2	0.93	2.55	3.282 (9)	135
C20—H20⋯O2^i^	0.93	2.50	3.384 (8)	159
C15—H15⋯*Cg*2^ii^	0.93	2.74	3.605 (6)	154
C21—H21⋯*Cg*1^iii^	0.93	2.86	3.731 (8)	156

Symmetry codes: (i) 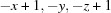; (ii) 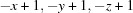; (iii) 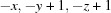.

**Table 2:** Hydrogen-bond geometry (Å, °) for (II) *Cg*1 is the centroid of the C14–C19 ring.

*D*—H⋯*A*	*D*—H	H⋯*A*	*D*⋯*A*	*D*—H⋯*A*
C5—H5⋯O2^i^	0.93	2.65	3.472 (3)	147
C12—H12*B*⋯*Cg*1^ii^	0.97	2.88	3.783 (3)	156

Symmetry codes: (i) 
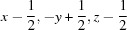
; (ii) 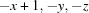.

**Table 3:** Experimental details

	(I)	(II)
Crystal data		
Chemical formula	C_21_H_16_O_2_S_2_	C_25_H_20_O_2_
*M*_r_	364.46	352.41
Crystal system, space group	Triclinic, *P* 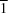	Monoclinic, *P*2_1_/*n*
Temperature (K)	296	296
*a*, *b*, *c* (Å)	7.1679 (11), 10.9915 (17), 11.2041 (16)	7.8610 (2), 16.7327 (5), 14.2260 (4)
α, β, γ (°)	75.491 (4), 83.148 (5), 86.424 (5)	90, 91.583 (2), 90
*V* (Å^3^)	848.0 (2)	1870.51 (9)
*Z*	2	4
Radiation type	Mo *K*α	Mo *K*α
μ (mm^−1^)	0.33	0.08
Crystal size (mm)	0.35 × 0.30 × 0.25	0.35 × 0.30 × 0.25

Data collection		
Diffractometer	Bruker APEXII CCD	Bruker APEXII CCD
Absorption correction	Multi-scan (*SADABS*; Bruker, 2008 ▸)	Multi-scan (*SADABS*; Bruker, 2008 ▸)
*T*_min_, *T*_max_	0.892, 0.922	0.973, 0.981
No. of measured, independent and observed [*I* > 2σ(*I*)] reflections	15428, 3661, 12676	16290, 4077, 2892
*R*_int_	0.000	0.030
(sin θ/λ)_max_ (Å^−1^)	0.639	0.639

Refinement		
*R*[*F* ^2^ > 2σ(*F* ^2^)], *wR*(*F* ^2^), *S*	0.064, 0.206, 1.08	0.048, 0.142, 1.02
No. of reflections	15439	4077
No. of parameters	305	266
No. of restraints	130	6
H-atom treatment	H-atom parameters constrained	H atoms treated by a mixture of independent and constrained refinement
Δρ_max_, Δρ_min_ (e Å^−3^)	0.38, −0.33	0.44, −0.26

Computer programs: *APEX2* and *SAINT* (Bruker, 2008 ▸), *SHELXS97* (Sheldrick, 2008 ▸), *SHELXL97* (Sheldrick, 2015 ▸), *ORTEP-3 for Windows* (Farrugia, 2012 ▸), *Mercury* (Macrae *et al.*, 2008 ▸) and *PLATON* (Spek, 2009 ▸).

## supplementary crystallographic information

### (I) 1,8-Bis(thiophen-2-yl)-14-oxatetracyclo[6.5.1.02,7.09,13]tetradeca-2(7),3,5-trien-10-one Crystal data

**Table:**

C_21_H_16_O_2_S_2_	*Z* = 2
*M**_r_* = 364.46	*F*(000) = 380
Triclinic, *P*1	*D*_x_ = 1.427 Mg m^−^^3^
Hall symbol: -P 1	Mo *K*α radiation, λ = 0.71073 Å
*a* = 7.1679 (11) Å	Cell parameters from 3661 reflections
*b* = 10.9915 (17) Å	θ = 1.9–27.0°
*c* = 11.2041 (16) Å	µ = 0.33 mm^−^^1^
α = 75.491 (4)°	*T* = 296 K
β = 83.148 (5)°	Block, colourless
γ = 86.424 (5)°	0.35 × 0.30 × 0.25 mm
*V* = 848.0 (2) Å^3^	

### (I) 1,8-Bis(thiophen-2-yl)-14-oxatetracyclo[6.5.1.02,7.09,13]tetradeca-2(7),3,5-trien-10-one Data collection

**Table:**

Bruker APEXII CCD diffractometer	3661 independent reflections
Radiation source: fine-focus sealed tube	12676 reflections with *I* > 2σ(*I*)
Graphite monochromator	*R*_int_ = 0.000
ω & φ scans	θ_max_ = 27.0°, θ_min_ = 1.9°
Absorption correction: multi-scan (*SADABS*; Bruker, 2008)	*h* = −9→9
*T*_min_ = 0.892, *T*_max_ = 0.922	*k* = −14→14
15428 measured reflections	*l* = −14→14

### (I) 1,8-Bis(thiophen-2-yl)-14-oxatetracyclo[6.5.1.02,7.09,13]tetradeca-2(7),3,5-trien-10-one Refinement

**Table:**

Refinement on *F*^2^	Secondary atom site location: difference Fourier map
Least-squares matrix: full	Hydrogen site location: inferred from neighbouring sites
*R*[*F*^2^ > 2σ(*F*^2^)] = 0.064	H-atom parameters constrained
*wR*(*F*^2^) = 0.206	*w* = 1/[σ^2^(*F*_o_^2^) + (0.1228*P*)^2^ + 0.3791*P*] where *P* = (*F*_o_^2^ + 2*F*_c_^2^)/3
*S* = 1.08	(Δ/σ)_max_ = 0.001
15439 reflections	Δρ_max_ = 0.38 e Å^−^^3^
305 parameters	Δρ_min_ = −0.33 e Å^−^^3^
130 restraints	Extinction correction: *SHELXL*, Fc^*^=kFc[1+0.001xFc^2^λ^3^/sin(2θ)]^-1/4^
Primary atom site location: structure-invariant direct methods	Extinction coefficient: 0.042 (4)

### (I) 1,8-Bis(thiophen-2-yl)-14-oxatetracyclo[6.5.1.02,7.09,13]tetradeca-2(7),3,5-trien-10-one Special details

**Table:**

Geometry. All e.s.d.'s (except the e.s.d. in the dihedral angle between two l.s. planes) are estimated using the full covariance matrix. The cell e.s.d.'s are taken into account individually in the estimation of e.s.d.'s in distances, angles and torsion angles; correlations between e.s.d.'s in cell parameters are only used when they are defined by crystal symmetry. An approximate (isotropic) treatment of cell e.s.d.'s is used for estimating e.s.d.'s involving l.s. planes.
Refinement. Refinement of *F*^2^ against ALL reflections. The weighted *R*-factor *wR* and goodness of fit *S* are based on *F*^2^, conventional *R*-factors *R* are based on *F*, with *F* set to zero for negative *F*^2^. The threshold expression of *F*^2^ > σ(*F*^2^) is used only for calculating *R*-factors(gt) *etc*. and is not relevant to the choice of reflections for refinement. *R*-factors based on *F*^2^ are statistically about twice as large as those based on *F*, and *R*- factors based on ALL data will be even larger.

### (I) 1,8-Bis(thiophen-2-yl)-14-oxatetracyclo[6.5.1.02,7.09,13]tetradeca-2(7),3,5-trien-10-one Fractional atomic coordinates and isotropic or equivalent isotropic displacement parameters (Å^2^)

**Table:**

	*x*	*y*	*z*	*U*_iso_*/*U*_eq_	Occ. (<1)
C1	0.2966 (2)	0.24768 (14)	0.80312 (15)	0.0320 (4)	
C2	0.2533 (3)	0.12787 (16)	0.87127 (18)	0.0427 (4)	
H2	0.2332	0.0645	0.8332	0.051*	
C3	0.2410 (3)	0.10627 (18)	1.00035 (19)	0.0512 (5)	
H3	0.2098	0.0270	1.0493	0.061*	
C4	0.2738 (3)	0.19886 (19)	1.05641 (18)	0.0503 (5)	
H4	0.2651	0.1813	1.1425	0.060*	
C5	0.3203 (2)	0.32007 (17)	0.98616 (15)	0.0408 (4)	
H5	0.3443	0.3830	1.0239	0.049*	
C6	0.3291 (2)	0.34209 (14)	0.85894 (14)	0.0316 (4)	
C7	0.3858 (2)	0.45424 (14)	0.75414 (14)	0.0303 (3)	
C8	0.5885 (2)	0.42773 (15)	0.69561 (15)	0.0341 (4)	
H8	0.6300	0.5003	0.6290	0.041*	
C9	0.5583 (2)	0.31589 (15)	0.64140 (15)	0.0343 (4)	
H9	0.6011	0.3340	0.5525	0.041*	
C10	0.3398 (2)	0.30417 (14)	0.66561 (14)	0.0313 (4)	
C13	0.7396 (3)	0.38703 (18)	0.78606 (18)	0.0465 (4)	
H13A	0.8594	0.4223	0.7482	0.056*	
H13B	0.7034	0.4151	0.8613	0.056*	
C12	0.7534 (3)	0.24352 (18)	0.81505 (19)	0.0506 (5)	
H12A	0.8837	0.2142	0.8171	0.061*	
H12B	0.6851	0.2077	0.8951	0.061*	
C11	0.6688 (3)	0.20498 (17)	0.71388 (19)	0.0439 (4)	
O1	0.27812 (15)	0.43350 (9)	0.65757 (10)	0.0315 (3)	
O2	0.6807 (2)	0.10143 (13)	0.69681 (16)	0.0662 (4)	
C14	0.3464 (2)	0.58158 (14)	0.77580 (15)	0.0331 (4)	
S1	0.14784 (9)	0.61494 (6)	0.86444 (6)	0.0503 (2)	0.901 (2)
C15	0.4516 (7)	0.6859 (4)	0.7309 (5)	0.0425 (9)	0.901 (2)
H15	0.5648	0.6863	0.6806	0.051*	0.901 (2)
C16	0.3716 (4)	0.7938 (3)	0.7684 (3)	0.0460 (7)	0.901 (2)
H16	0.4258	0.8721	0.7460	0.055*	0.901 (2)
C17	0.2080 (4)	0.7687 (2)	0.8404 (3)	0.0466 (7)	0.901 (2)
H17	0.1354	0.8280	0.8735	0.056*	0.901 (2)
S1'	0.483 (2)	0.7018 (12)	0.7053 (15)	0.062 (3)	0.099 (2)
C15'	0.216 (3)	0.5936 (18)	0.8800 (16)	0.0425 (9)	0.099 (2)
H15'	0.1643	0.5290	0.9438	0.051*	0.099 (2)
C16'	0.181 (4)	0.7267 (17)	0.865 (3)	0.039 (4)	0.099 (2)
H16'	0.0845	0.7639	0.9085	0.047*	0.099 (2)
C17'	0.319 (3)	0.793 (3)	0.772 (3)	0.042 (5)	0.099 (2)
H17'	0.3205	0.8799	0.7489	0.051*	0.099 (2)
S2	0.0599 (2)	0.3235 (2)	0.50935 (18)	0.0465 (4)	0.666 (2)
C18	0.2491 (7)	0.2522 (5)	0.5765 (5)	0.0325 (12)	0.666 (2)
C19	0.3072 (13)	0.1376 (7)	0.5464 (7)	0.056 (2)	0.666 (2)
H19	0.4085	0.0855	0.5742	0.067*	0.666 (2)
C20	0.1796 (9)	0.1168 (7)	0.4643 (6)	0.0585 (14)	0.666 (2)
H20	0.1901	0.0457	0.4328	0.070*	0.666 (2)
C21	0.0425 (11)	0.2074 (5)	0.4352 (7)	0.0541 (14)	0.666 (2)
H21	−0.0478	0.2061	0.3824	0.065*	0.666 (2)
S2'	0.2950 (8)	0.1143 (4)	0.5432 (5)	0.0558 (9)	0.334 (2)
C18'	0.2398 (16)	0.2505 (10)	0.5834 (11)	0.047 (3)	0.334 (2)
C19'	0.0888 (17)	0.3156 (14)	0.5201 (12)	0.054 (3)	0.334 (2)
H19'	0.0359	0.3928	0.5289	0.065*	0.334 (2)
C20'	0.030 (2)	0.2443 (11)	0.4405 (13)	0.052 (3)	0.334 (2)
H20'	−0.0670	0.2724	0.3911	0.062*	0.334 (2)
C21'	0.1282 (16)	0.1300 (14)	0.4422 (11)	0.047 (2)	0.334 (2)
H21'	0.1070	0.0730	0.3968	0.057*	0.334 (2)

### (I) 1,8-Bis(thiophen-2-yl)-14-oxatetracyclo[6.5.1.02,7.09,13]tetradeca-2(7),3,5-trien-10-one Atomic displacement parameters (Å^2^)

**Table:**

	*U*^11^	*U*^22^	*U*^33^	*U*^12^	*U*^13^	*U*^23^
C1	0.0279 (8)	0.0281 (8)	0.0361 (9)	0.0034 (6)	−0.0017 (6)	−0.0028 (7)
C2	0.0422 (10)	0.0311 (9)	0.0500 (11)	−0.0004 (7)	0.0003 (8)	−0.0039 (8)
C3	0.0519 (12)	0.0392 (10)	0.0481 (11)	−0.0016 (9)	0.0018 (9)	0.0128 (9)
C4	0.0492 (11)	0.0563 (12)	0.0337 (9)	0.0028 (9)	0.0014 (8)	0.0070 (9)
C5	0.0453 (10)	0.0435 (10)	0.0306 (9)	0.0000 (8)	−0.0037 (7)	−0.0039 (8)
C6	0.0274 (8)	0.0333 (8)	0.0313 (8)	0.0014 (6)	−0.0012 (6)	−0.0043 (7)
C7	0.0335 (8)	0.0287 (8)	0.0280 (8)	−0.0007 (6)	−0.0042 (6)	−0.0052 (6)
C8	0.0326 (9)	0.0304 (8)	0.0357 (9)	−0.0016 (7)	0.0012 (7)	−0.0037 (7)
C9	0.0369 (9)	0.0310 (8)	0.0325 (8)	−0.0008 (7)	0.0020 (7)	−0.0061 (7)
C10	0.0356 (9)	0.0229 (7)	0.0327 (8)	0.0015 (6)	−0.0034 (7)	−0.0025 (6)
C13	0.0374 (10)	0.0515 (11)	0.0514 (11)	0.0012 (8)	−0.0059 (8)	−0.0142 (9)
C12	0.0412 (11)	0.0531 (12)	0.0532 (12)	0.0095 (9)	−0.0098 (9)	−0.0055 (9)
C11	0.0364 (10)	0.0370 (10)	0.0531 (11)	0.0045 (7)	0.0094 (8)	−0.0090 (8)
O1	0.0387 (6)	0.0243 (5)	0.0313 (6)	0.0015 (4)	−0.0071 (5)	−0.0056 (4)
O2	0.0718 (10)	0.0432 (8)	0.0817 (11)	0.0155 (7)	−0.0040 (8)	−0.0182 (8)
C14	0.0363 (9)	0.0294 (8)	0.0326 (8)	0.0028 (7)	−0.0051 (7)	−0.0062 (7)
S1	0.0510 (4)	0.0399 (3)	0.0587 (4)	0.0006 (3)	0.0117 (3)	−0.0180 (3)
C15	0.0459 (18)	0.0312 (17)	0.048 (2)	−0.0016 (12)	−0.0048 (16)	−0.0055 (14)
C16	0.0471 (16)	0.0316 (12)	0.0608 (16)	−0.0015 (12)	−0.0115 (13)	−0.0114 (11)
C17	0.0575 (17)	0.0288 (13)	0.0560 (17)	0.0017 (13)	−0.0049 (13)	−0.0167 (12)
S1'	0.092 (7)	0.034 (4)	0.057 (6)	−0.014 (4)	0.010 (4)	−0.013 (4)
C15'	0.0459 (18)	0.0312 (17)	0.048 (2)	−0.0016 (12)	−0.0048 (16)	−0.0055 (14)
C16'	0.044 (7)	0.027 (6)	0.050 (8)	−0.011 (6)	−0.012 (6)	−0.011 (6)
C17'	0.044 (10)	0.024 (7)	0.059 (10)	−0.007 (7)	−0.011 (8)	−0.005 (7)
S2	0.0436 (5)	0.0523 (7)	0.0469 (7)	−0.0053 (5)	−0.0108 (5)	−0.0146 (5)
C18	0.036 (2)	0.033 (3)	0.029 (2)	−0.009 (2)	0.0018 (18)	−0.010 (2)
C19	0.073 (3)	0.056 (4)	0.044 (3)	−0.010 (3)	−0.007 (2)	−0.020 (3)
C20	0.081 (4)	0.052 (2)	0.052 (3)	−0.027 (2)	0.000 (2)	−0.028 (2)
C21	0.060 (3)	0.060 (3)	0.055 (2)	−0.022 (3)	−0.0015 (19)	−0.036 (3)
S2'	0.0763 (18)	0.0347 (12)	0.0595 (18)	−0.0008 (11)	−0.0097 (14)	−0.0163 (10)
C18'	0.071 (6)	0.029 (5)	0.041 (6)	0.004 (5)	−0.007 (5)	−0.006 (4)
C19'	0.079 (7)	0.051 (5)	0.044 (5)	−0.007 (4)	−0.017 (4)	−0.028 (4)
C20'	0.059 (4)	0.060 (6)	0.043 (4)	−0.006 (4)	−0.009 (3)	−0.023 (4)
C21'	0.055 (6)	0.056 (5)	0.041 (5)	−0.022 (4)	−0.003 (4)	−0.026 (4)

### (I) 1,8-Bis(thiophen-2-yl)-14-oxatetracyclo[6.5.1.02,7.09,13]tetradeca-2(7),3,5-trien-10-one Geometric parameters (Å, º)

**Table:**

C1—C2	1.380 (2)	C14—C15'	1.435 (10)
C1—C6	1.383 (2)	C14—S1'	1.667 (9)
C1—C10	1.512 (2)	C14—S1	1.7109 (17)
C2—C3	1.398 (3)	S1—C17	1.718 (2)
C2—H2	0.9300	C15—C16	1.421 (5)
C3—C4	1.368 (3)	C15—H15	0.9300
C3—H3	0.9300	C16—C17	1.343 (3)
C4—C5	1.405 (2)	C16—H16	0.9300
C4—H4	0.9300	C17—H17	0.9300
C5—C6	1.379 (2)	S1'—C17'	1.715 (10)
C5—H5	0.9300	C15'—C16'	1.440 (10)
C6—C7	1.512 (2)	C15'—H15'	0.9300
C7—O1	1.4709 (18)	C16'—C17'	1.437 (10)
C7—C14	1.484 (2)	C16'—H16'	0.9300
C7—C8	1.562 (2)	C17'—H17'	0.9300
C8—C9	1.538 (2)	S2—C18	1.684 (5)
C8—C13	1.541 (3)	S2—C21	1.706 (5)
C8—H8	0.9800	C18—C19	1.410 (7)
C9—C11	1.520 (2)	C19—C20	1.437 (8)
C9—C10	1.565 (2)	C19—H19	0.9300
C9—H9	0.9800	C20—C21	1.362 (6)
C10—O1	1.4462 (17)	C20—H20	0.9300
C10—C18'	1.481 (6)	C21—H21	0.9300
C10—C18	1.494 (3)	S2'—C18'	1.680 (7)
C13—C12	1.528 (2)	S2'—C21'	1.714 (8)
C13—H13A	0.9700	C18'—C19'	1.420 (9)
C13—H13B	0.9700	C19'—C20'	1.438 (9)
C12—C11	1.506 (3)	C19'—H19'	0.9300
C12—H12A	0.9700	C20'—C21'	1.399 (9)
C12—H12B	0.9700	C20'—H20'	0.9300
C11—O2	1.197 (2)	C21'—H21'	0.9300
C14—C15	1.365 (4)		
			
C2—C1—C6	121.96 (16)	O2—C11—C9	125.42 (19)
C2—C1—C10	132.73 (15)	C12—C11—C9	109.52 (15)
C6—C1—C10	104.99 (13)	C10—O1—C7	97.08 (11)
C1—C2—C3	116.87 (17)	C15—C14—C15'	111.7 (9)
C1—C2—H2	121.6	C15—C14—C7	128.0 (3)
C3—C2—H2	121.6	C15'—C14—C7	118.0 (8)
C4—C3—C2	121.60 (17)	C15'—C14—S1'	118.7 (10)
C4—C3—H3	119.2	C7—C14—S1'	121.8 (6)
C2—C3—H3	119.2	C15—C14—S1	110.6 (3)
C3—C4—C5	121.10 (17)	C7—C14—S1	121.36 (12)
C3—C4—H4	119.5	S1'—C14—S1	116.7 (6)
C5—C4—H4	119.5	C14—S1—C17	91.77 (12)
C6—C5—C4	117.33 (16)	C14—C15—C16	113.5 (4)
C6—C5—H5	121.3	C14—C15—H15	123.3
C4—C5—H5	121.3	C16—C15—H15	123.3
C5—C6—C1	121.12 (15)	C17—C16—C15	111.7 (4)
C5—C6—C7	132.87 (15)	C17—C16—H16	124.2
C1—C6—C7	105.81 (13)	C15—C16—H16	124.2
O1—C7—C14	111.68 (12)	C16—C17—S1	112.4 (3)
O1—C7—C6	100.32 (11)	C16—C17—H17	123.8
C14—C7—C6	118.01 (13)	S1—C17—H17	123.8
O1—C7—C8	99.09 (12)	C14—S1'—C17'	85.9 (13)
C14—C7—C8	116.34 (13)	C14—C15'—C16'	105.4 (17)
C6—C7—C8	108.61 (12)	C14—C15'—H15'	127.3
C9—C8—C13	107.84 (13)	C16'—C15'—H15'	127.3
C9—C8—C7	101.82 (12)	C17'—C16'—C15'	109 (3)
C13—C8—C7	116.37 (14)	C17'—C16'—H16'	125.6
C9—C8—H8	110.1	C15'—C16'—H16'	125.6
C13—C8—H8	110.1	C16'—C17'—S1'	117 (2)
C7—C8—H8	110.1	C16'—C17'—H17'	121.7
C11—C9—C8	106.05 (14)	S1'—C17'—H17'	121.7
C11—C9—C10	114.47 (13)	C18—S2—C21	92.4 (3)
C8—C9—C10	101.93 (12)	C19—C18—C10	123.5 (5)
C11—C9—H9	111.3	C19—C18—S2	114.5 (5)
C8—C9—H9	111.3	C10—C18—S2	122.0 (3)
C10—C9—H9	111.3	C18—C19—C20	106.7 (8)
O1—C10—C18'	110.3 (4)	C18—C19—H19	126.6
O1—C10—C18	110.6 (2)	C20—C19—H19	126.6
O1—C10—C1	100.81 (12)	C21—C20—C19	116.1 (7)
C18'—C10—C1	115.8 (5)	C21—C20—H20	121.9
C18—C10—C1	118.9 (3)	C19—C20—H20	121.9
O1—C10—C9	101.51 (11)	C20—C21—S2	110.3 (6)
C18'—C10—C9	119.8 (5)	C20—C21—H21	124.8
C18—C10—C9	116.4 (2)	S2—C21—H21	124.8
C1—C10—C9	106.12 (13)	C18'—S2'—C21'	96.1 (7)
C12—C13—C8	106.01 (14)	C19'—C18'—C10	122.6 (8)
C12—C13—H13A	110.5	C19'—C18'—S2'	110.9 (8)
C8—C13—H13A	110.5	C10—C18'—S2'	126.3 (7)
C12—C13—H13B	110.5	C18'—C19'—C20'	109.8 (13)
C8—C13—H13B	110.5	C18'—C19'—H19'	125.1
H13A—C13—H13B	108.7	C20'—C19'—H19'	125.1
C11—C12—C13	107.02 (16)	C21'—C20'—C19'	115.6 (15)
C11—C12—H12A	110.3	C21'—C20'—H20'	122.2
C13—C12—H12A	110.3	C19'—C20'—H20'	122.2
C11—C12—H12B	110.3	C20'—C21'—S2'	107.5 (12)
C13—C12—H12B	110.3	C20'—C21'—H21'	126.3
H12A—C12—H12B	108.6	S2'—C21'—H21'	126.3
O2—C11—C12	124.99 (19)		
			
C6—C1—C2—C3	0.9 (2)	O1—C7—C14—C15'	99.5 (11)
C10—C1—C2—C3	173.29 (18)	C6—C7—C14—C15'	−15.9 (11)
C1—C2—C3—C4	−1.2 (3)	C8—C7—C14—C15'	−147.8 (11)
C2—C3—C4—C5	0.3 (3)	O1—C7—C14—S1'	−95.0 (8)
C3—C4—C5—C6	0.8 (3)	C6—C7—C14—S1'	149.6 (8)
C4—C5—C6—C1	−1.1 (2)	C8—C7—C14—S1'	17.8 (8)
C4—C5—C6—C7	−175.18 (16)	O1—C7—C14—S1	79.51 (16)
C2—C1—C6—C5	0.3 (2)	C6—C7—C14—S1	−35.94 (19)
C10—C1—C6—C5	−173.95 (15)	C8—C7—C14—S1	−167.75 (12)
C2—C1—C6—C7	175.76 (15)	C15—C14—S1—C17	0.0 (3)
C10—C1—C6—C7	1.51 (16)	C15'—C14—S1—C17	97 (3)
C5—C6—C7—O1	−154.30 (17)	C7—C14—S1—C17	−179.14 (16)
C1—C6—C7—O1	31.01 (15)	S1'—C14—S1—C17	−4.4 (8)
C5—C6—C7—C14	−32.8 (2)	C15'—C14—C15—C16	−18.8 (11)
C1—C6—C7—C14	152.48 (14)	C7—C14—C15—C16	179.1 (2)
C5—C6—C7—C8	102.4 (2)	S1'—C14—C15—C16	147 (7)
C1—C6—C7—C8	−72.33 (15)	S1—C14—C15—C16	0.1 (4)
O1—C7—C8—C9	−39.30 (14)	C14—C15—C16—C17	−0.1 (3)
C14—C7—C8—C9	−159.08 (13)	C15—C16—C17—S1	0.14 (18)
C6—C7—C8—C9	64.90 (15)	C14—S1—C17—C16	−0.08 (19)
O1—C7—C8—C13	−156.24 (13)	C15—C14—S1'—C17'	−35 (7)
C14—C7—C8—C13	83.98 (18)	C15'—C14—S1'—C17'	−20.5 (18)
C6—C7—C8—C13	−52.04 (17)	C7—C14—S1'—C17'	174.1 (10)
C13—C8—C9—C11	8.25 (17)	S1—C14—S1'—C17'	−0.6 (12)
C7—C8—C9—C11	−114.71 (14)	C15—C14—C15'—C16'	25.8 (18)
C13—C8—C9—C10	128.33 (14)	C7—C14—C15'—C16'	−170.2 (12)
C7—C8—C9—C10	5.38 (15)	S1'—C14—C15'—C16'	24 (2)
C2—C1—C10—O1	152.40 (17)	S1—C14—C15'—C16'	−64 (2)
C6—C1—C10—O1	−34.24 (15)	C14—C15'—C16'—C17'	−13.3 (12)
C2—C1—C10—C18'	33.4 (5)	C15'—C16'—C17'—S1'	0.0 (2)
C6—C1—C10—C18'	−153.2 (5)	C14—S1'—C17'—C16'	11.0 (10)
C2—C1—C10—C18	31.4 (3)	O1—C10—C18—C19	167.7 (3)
C6—C1—C10—C18	−155.2 (3)	C18'—C10—C18—C19	−106 (10)
C2—C1—C10—C9	−102.1 (2)	C1—C10—C18—C19	−76.4 (4)
C6—C1—C10—C9	71.22 (15)	C9—C10—C18—C19	52.5 (4)
C11—C9—C10—O1	144.99 (13)	O1—C10—C18—S2	−15.6 (4)
C8—C9—C10—O1	30.99 (14)	C18'—C10—C18—S2	71 (10)
C11—C9—C10—C18'	−93.4 (6)	C1—C10—C18—S2	100.3 (4)
C8—C9—C10—C18'	152.7 (6)	C9—C10—C18—S2	−130.7 (3)
C11—C9—C10—C18	−94.9 (3)	C21—S2—C18—C19	−0.18 (14)
C8—C9—C10—C18	151.1 (3)	C21—S2—C18—C10	−177.2 (5)
C11—C9—C10—C1	40.03 (17)	C10—C18—C19—C20	176.7 (6)
C8—C9—C10—C1	−73.96 (14)	S2—C18—C19—C20	−0.26 (18)
C9—C8—C13—C12	−16.94 (19)	C18—C19—C20—C21	0.7 (4)
C7—C8—C13—C12	96.62 (17)	C19—C20—C21—S2	−0.9 (4)
C8—C13—C12—C11	19.1 (2)	C18—S2—C21—C20	0.6 (3)
C13—C12—C11—O2	168.38 (19)	O1—C10—C18'—C19'	−7.4 (8)
C13—C12—C11—C9	−14.4 (2)	C18—C10—C18'—C19'	−102 (10)
C8—C9—C11—O2	−179.04 (18)	C1—C10—C18'—C19'	106.2 (6)
C10—C9—C11—O2	69.4 (2)	C9—C10—C18'—C19'	−124.6 (5)
C8—C9—C11—C12	3.79 (18)	O1—C10—C18'—S2'	167.7 (7)
C10—C9—C11—C12	−107.75 (17)	C18—C10—C18'—S2'	73 (10)
C18'—C10—O1—C7	175.2 (6)	C1—C10—C18'—S2'	−78.6 (10)
C18—C10—O1—C7	179.1 (3)	C9—C10—C18'—S2'	50.5 (11)
C1—C10—O1—C7	52.34 (13)	C21'—S2'—C18'—C19'	−0.10 (15)
C9—C10—O1—C7	−56.77 (13)	C21'—S2'—C18'—C10	−175.7 (12)
C14—C7—O1—C10	−177.00 (13)	C10—C18'—C19'—C20'	175.6 (12)
C6—C7—O1—C10	−51.13 (13)	S2'—C18'—C19'—C20'	−0.16 (19)
C8—C7—O1—C10	59.83 (12)	C18'—C19'—C20'—C21'	0.4 (4)
O1—C7—C14—C15	−99.5 (3)	C19'—C20'—C21'—S2'	−0.5 (4)
C6—C7—C14—C15	145.1 (3)	C18'—S2'—C21'—C20'	0.3 (3)
C8—C7—C14—C15	13.3 (4)		

### (I) 1,8-Bis(thiophen-2-yl)-14-oxatetracyclo[6.5.1.02,7.09,13]tetradeca-2(7),3,5-trien-10-one Hydrogen-bond geometry (Å, º)

Cg1 and Cg2 are the centroids of the S1,C14–C17 and S2/C18–C21 rings, respectively.

**Table:**

*D*—H···*A*	*D*—H	H···*A*	*D*···*A*	*D*—H···*A*
C19—H19···O2	0.93	2.55	3.282 (9)	135
C20—H20···O2^i^	0.93	2.50	3.384 (8)	159
C15—H15···*Cg*2^ii^	0.93	2.74	3.605 (6)	154
C21—H21···*Cg*1^iii^	0.93	2.86	3.731 (8)	156

Symmetry codes: (i) −*x*+1, −*y*, −*z*+1; (ii) −*x*+1, −*y*+1, −*z*+1; (iii) −*x*, −*y*+1, −*z*+1.

### (II) 1,8-Diphenyl-14-oxatetracyclo[6.5.1.02,7.09,13] tetradeca-2,4,6-trien-10-one Crystal data

**Table:**

C_25_H_20_O_2_	*F*(000) = 744
*M**_r_* = 352.41	*D*_x_ = 1.251 Mg m^−^^3^
Monoclinic, *P*2_1_/*n*	Mo *K*α radiation, λ = 0.71073 Å
Hall symbol: -P 2yn	Cell parameters from 4077 reflections
*a* = 7.8610 (2) Å	θ = 1.9–27.0°
*b* = 16.7327 (5) Å	µ = 0.08 mm^−^^1^
*c* = 14.2260 (4) Å	*T* = 296 K
β = 91.583 (2)°	Block, colourless
*V* = 1870.51 (9) Å^3^	0.35 × 0.30 × 0.25 mm
*Z* = 4	

### (II) 1,8-Diphenyl-14-oxatetracyclo[6.5.1.02,7.09,13] tetradeca-2,4,6-trien-10-one Data collection

**Table:**

Bruker APEXII CCD diffractometer	4077 independent reflections
Radiation source: fine-focus sealed tube	2892 reflections with *I* > 2σ(*I*)
Graphite monochromator	*R*_int_ = 0.030
ω & φ scans	θ_max_ = 27.0°, θ_min_ = 1.9°
Absorption correction: multi-scan (*SADABS*; Bruker, 2008)	*h* = −9→10
*T*_min_ = 0.973, *T*_max_ = 0.981	*k* = −19→21
16290 measured reflections	*l* = −18→18

### (II) 1,8-Diphenyl-14-oxatetracyclo[6.5.1.02,7.09,13] tetradeca-2,4,6-trien-10-one Refinement

**Table:**

Refinement on *F*^2^	Primary atom site location: structure-invariant direct methods
Least-squares matrix: full	Secondary atom site location: difference Fourier map
*R*[*F*^2^ > 2σ(*F*^2^)] = 0.048	Hydrogen site location: inferred from neighbouring sites
*wR*(*F*^2^) = 0.142	H atoms treated by a mixture of independent and constrained refinement
*S* = 1.02	*w* = 1/[σ^2^(*F*_o_^2^) + (0.0536*P*)^2^ + 0.6324*P*] where *P* = (*F*_o_^2^ + 2*F*_c_^2^)/3
4077 reflections	(Δ/σ)_max_ < 0.001
266 parameters	Δρ_max_ = 0.44 e Å^−^^3^
6 restraints	Δρ_min_ = −0.26 e Å^−^^3^

### (II) 1,8-Diphenyl-14-oxatetracyclo[6.5.1.02,7.09,13] tetradeca-2,4,6-trien-10-one Special details

**Table:**

Geometry. All e.s.d.'s (except the e.s.d. in the dihedral angle between two l.s. planes) are estimated using the full covariance matrix. The cell e.s.d.'s are taken into account individually in the estimation of e.s.d.'s in distances, angles and torsion angles; correlations between e.s.d.'s in cell parameters are only used when they are defined by crystal symmetry. An approximate (isotropic) treatment of cell e.s.d.'s is used for estimating e.s.d.'s involving l.s. planes.
Refinement. Refinement of *F*^2^ against ALL reflections. The weighted *R*-factor *wR* and goodness of fit *S* are based on *F*^2^, conventional *R*-factors *R* are based on *F*, with *F* set to zero for negative *F*^2^. The threshold expression of *F*^2^ > σ(*F*^2^) is used only for calculating *R*-factors(gt) *etc*. and is not relevant to the choice of reflections for refinement. *R*-factors based on *F*^2^ are statistically about twice as large as those based on *F*, and *R*- factors based on ALL data will be even larger.

### (II) 1,8-Diphenyl-14-oxatetracyclo[6.5.1.02,7.09,13] tetradeca-2,4,6-trien-10-one Fractional atomic coordinates and isotropic or equivalent isotropic displacement parameters (Å^2^)

**Table:**

	*x*	*y*	*z*	*U*_iso_*/*U*_eq_	Occ. (<1)
C5	0.1799 (2)	0.33834 (11)	0.02163 (16)	0.0658 (5)	
H5	0.1817	0.3370	−0.0437	0.079*	
C4	0.1426 (3)	0.40825 (12)	0.0699 (2)	0.0815 (7)	
H4	0.1182	0.4546	0.0361	0.098*	
C3	0.1410 (3)	0.41030 (12)	0.1663 (2)	0.0841 (7)	
H3	0.1149	0.4579	0.1963	0.101*	
C2	0.1774 (2)	0.34310 (11)	0.21975 (17)	0.0680 (5)	
H2	0.1772	0.3447	0.2851	0.082*	
C1	0.2140 (2)	0.27359 (10)	0.17223 (13)	0.0525 (4)	
C6	0.2141 (2)	0.27118 (9)	0.07483 (13)	0.0514 (4)	
C7	0.2701 (2)	0.18689 (9)	0.05029 (12)	0.0458 (4)	
C8	0.4653 (2)	0.18451 (10)	0.07563 (12)	0.0502 (4)	
H8	0.5242	0.2310	0.0501	0.060*	
C9	0.4652 (2)	0.18675 (9)	0.18437 (12)	0.0493 (4)	
H9	0.5257	0.2337	0.2093	0.059*	
C10	0.2700 (2)	0.19076 (9)	0.20431 (12)	0.0472 (4)	
C20	0.2116 (2)	0.16250 (10)	0.29781 (12)	0.0526 (4)	
C25	0.0743 (3)	0.11180 (11)	0.30508 (14)	0.0629 (5)	
H25	0.0179	0.0937	0.2509	0.075*	
C24	0.0199 (3)	0.08766 (13)	0.39196 (16)	0.0801 (7)	
H24	−0.0735	0.0539	0.3960	0.096*	
C23	0.1031 (4)	0.11334 (15)	0.47233 (17)	0.0852 (7)	
H23	0.0671	0.0964	0.5308	0.102*	
C22	0.2397 (3)	0.16412 (17)	0.46657 (15)	0.0852 (7)	
H22	0.2960	0.1816	0.5211	0.102*	
C21	0.2937 (3)	0.18934 (14)	0.37959 (14)	0.0722 (6)	
H21	0.3851	0.2244	0.3759	0.087*	
C14	0.2107 (2)	0.15404 (9)	−0.04292 (12)	0.0488 (4)	
C19	0.0741 (2)	0.10191 (11)	−0.05016 (13)	0.0571 (4)	
H19	0.0197	0.0856	0.0038	0.069*	
C18	0.0180 (3)	0.07400 (12)	−0.13688 (14)	0.0695 (5)	
H18	−0.0738	0.0390	−0.1409	0.083*	
C17	0.0967 (3)	0.09743 (13)	−0.21708 (14)	0.0713 (6)	
H17	0.0585	0.0784	−0.2753	0.086*	
C16	0.2318 (3)	0.14902 (13)	−0.21109 (14)	0.0716 (6)	
H16	0.2854	0.1649	−0.2655	0.086*	
C15	0.2892 (3)	0.17770 (12)	−0.12477 (13)	0.0626 (5)	
H15	0.3806	0.2130	−0.1214	0.075*	
O1	0.20140 (13)	0.14227 (6)	0.12775 (7)	0.0462 (3)	
C11	0.5511 (3)	0.10887 (12)	0.21700 (14)	0.0602 (5)	
C12	0.5542 (4)	0.05380 (14)	0.13504 (15)	0.0900 (8)	
H12A	0.4549	0.0193	0.1339	0.108*	
H12B	0.6556	0.0208	0.1379	0.108*	
C13	0.5538 (3)	0.10651 (13)	0.04969 (14)	0.0639 (5)	
O2	0.5955 (4)	0.09193 (17)	0.29524 (17)	0.0767 (10)	0.579 (4)
O2'	0.6160 (5)	0.0954 (2)	−0.0248 (3)	0.0793 (14)	0.421 (4)
H13A	0.505 (5)	0.080 (2)	−0.0058 (19)	0.095*	0.579 (4)
H13B	0.6752 (15)	0.113 (2)	0.044 (3)	0.095*	0.579 (4)
H11A	0.662 (3)	0.129 (3)	0.238 (4)	0.095*	0.421 (4)
H11B	0.498 (7)	0.086 (3)	0.272 (2)	0.095*	0.421 (4)

### (II) 1,8-Diphenyl-14-oxatetracyclo[6.5.1.02,7.09,13] tetradeca-2,4,6-trien-10-one Atomic displacement parameters (Å^2^)

**Table:**

	*U*^11^	*U*^22^	*U*^33^	*U*^12^	*U*^13^	*U*^23^
C5	0.0512 (10)	0.0493 (10)	0.0966 (15)	0.0025 (8)	−0.0028 (10)	0.0124 (10)
C4	0.0635 (13)	0.0455 (11)	0.135 (2)	0.0101 (9)	−0.0049 (13)	0.0116 (12)
C3	0.0682 (14)	0.0482 (12)	0.136 (2)	0.0126 (10)	0.0028 (14)	−0.0180 (13)
C2	0.0527 (11)	0.0538 (11)	0.0975 (15)	0.0043 (8)	0.0039 (10)	−0.0190 (10)
C1	0.0395 (8)	0.0436 (9)	0.0745 (12)	0.0005 (7)	0.0014 (8)	−0.0080 (8)
C6	0.0384 (8)	0.0418 (9)	0.0739 (12)	0.0000 (6)	0.0008 (7)	0.0016 (8)
C7	0.0424 (8)	0.0382 (8)	0.0568 (9)	−0.0018 (6)	0.0035 (7)	0.0041 (7)
C8	0.0432 (9)	0.0430 (9)	0.0645 (10)	−0.0013 (7)	0.0044 (7)	0.0030 (7)
C9	0.0414 (9)	0.0424 (8)	0.0640 (10)	−0.0015 (6)	−0.0011 (7)	−0.0076 (7)
C10	0.0425 (8)	0.0422 (8)	0.0567 (9)	−0.0024 (6)	−0.0004 (7)	−0.0088 (7)
C20	0.0506 (10)	0.0519 (10)	0.0553 (10)	0.0085 (7)	0.0036 (8)	−0.0076 (8)
C25	0.0728 (13)	0.0538 (11)	0.0626 (11)	−0.0048 (9)	0.0116 (9)	−0.0071 (9)
C24	0.0997 (18)	0.0633 (13)	0.0789 (15)	−0.0023 (11)	0.0314 (13)	0.0009 (11)
C23	0.104 (2)	0.0887 (17)	0.0645 (13)	0.0329 (15)	0.0241 (13)	0.0103 (12)
C22	0.0789 (16)	0.121 (2)	0.0553 (12)	0.0354 (15)	−0.0007 (11)	−0.0150 (12)
C21	0.0566 (11)	0.0944 (16)	0.0655 (12)	0.0067 (10)	0.0003 (9)	−0.0165 (11)
C14	0.0484 (9)	0.0442 (9)	0.0539 (9)	0.0052 (7)	0.0007 (7)	0.0049 (7)
C19	0.0592 (11)	0.0538 (10)	0.0581 (10)	−0.0036 (8)	−0.0017 (8)	0.0008 (8)
C18	0.0728 (13)	0.0664 (12)	0.0684 (13)	−0.0021 (10)	−0.0132 (10)	−0.0056 (10)
C17	0.0818 (15)	0.0737 (13)	0.0576 (11)	0.0183 (11)	−0.0129 (10)	−0.0064 (10)
C16	0.0791 (14)	0.0835 (15)	0.0524 (11)	0.0246 (12)	0.0061 (10)	0.0137 (10)
C15	0.0590 (11)	0.0667 (12)	0.0624 (11)	0.0037 (9)	0.0040 (9)	0.0114 (9)
O1	0.0462 (6)	0.0423 (6)	0.0501 (6)	−0.0068 (5)	0.0018 (5)	−0.0020 (5)
C11	0.0576 (11)	0.0595 (11)	0.0632 (12)	0.0123 (9)	−0.0045 (9)	−0.0026 (9)
C12	0.126 (2)	0.0669 (14)	0.0768 (14)	0.0412 (14)	−0.0065 (13)	−0.0058 (11)
C13	0.0594 (12)	0.0681 (12)	0.0642 (12)	0.0189 (9)	0.0037 (9)	−0.0051 (9)
O2	0.0843 (19)	0.0811 (18)	0.0637 (16)	0.0222 (14)	−0.0165 (13)	−0.0040 (12)
O2'	0.074 (2)	0.089 (3)	0.075 (3)	0.0169 (19)	0.0201 (19)	−0.0083 (19)

### (II) 1,8-Diphenyl-14-oxatetracyclo[6.5.1.02,7.09,13] tetradeca-2,4,6-trien-10-one Geometric parameters (Å, º)

**Table:**

C5—C6	1.377 (2)	C23—H23	0.9300
C5—C4	1.392 (3)	C22—C21	1.385 (3)
C5—H5	0.9300	C22—H22	0.9300
C4—C3	1.372 (4)	C21—H21	0.9300
C4—H4	0.9300	C14—C19	1.385 (2)
C3—C2	1.383 (3)	C14—C15	1.390 (2)
C3—H3	0.9300	C19—C18	1.380 (3)
C2—C1	1.380 (2)	C19—H19	0.9300
C2—H2	0.9300	C18—C17	1.370 (3)
C1—C6	1.386 (3)	C18—H18	0.9300
C1—C10	1.520 (2)	C17—C16	1.370 (3)
C6—C7	1.521 (2)	C17—H17	0.9300
C7—O1	1.4480 (19)	C16—C15	1.382 (3)
C7—C14	1.498 (2)	C16—H16	0.9300
C7—C8	1.567 (2)	C15—H15	0.9300
C8—C13	1.529 (2)	C11—O2	1.191 (3)
C8—C9	1.547 (2)	C11—C12	1.487 (3)
C8—H8	0.9800	C11—H11A	0.975 (10)
C9—C11	1.534 (2)	C11—H11B	0.975 (10)
C9—C10	1.570 (2)	C12—C13	1.501 (3)
C9—H9	0.9800	C12—H12A	0.9700
C10—O1	1.4500 (18)	C12—H12B	0.9700
C10—C20	1.496 (2)	C13—O2'	1.194 (4)
C20—C25	1.379 (3)	C13—H13A	0.973 (10)
C20—C21	1.389 (3)	C13—H13B	0.967 (10)
C25—C24	1.380 (3)	O2—H11A	1.16 (5)
C25—H25	0.9300	O2—H11B	0.83 (5)
C24—C23	1.371 (3)	O2'—H13A	0.95 (4)
C24—H24	0.9300	O2'—H13B	1.11 (4)
C23—C22	1.374 (4)		
			
C6—C5—C4	117.1 (2)	C23—C22—H22	120.0
C6—C5—H5	121.5	C21—C22—H22	120.0
C4—C5—H5	121.5	C22—C21—C20	120.2 (2)
C3—C4—C5	121.5 (2)	C22—C21—H21	119.9
C3—C4—H4	119.3	C20—C21—H21	119.9
C5—C4—H4	119.3	C19—C14—C15	118.56 (17)
C4—C3—C2	121.4 (2)	C19—C14—C7	121.26 (15)
C4—C3—H3	119.3	C15—C14—C7	120.14 (16)
C2—C3—H3	119.3	C18—C19—C14	120.48 (18)
C1—C2—C3	117.3 (2)	C18—C19—H19	119.8
C1—C2—H2	121.3	C14—C19—H19	119.8
C3—C2—H2	121.3	C17—C18—C19	120.5 (2)
C2—C1—C6	121.34 (17)	C17—C18—H18	119.7
C2—C1—C10	133.16 (18)	C19—C18—H18	119.7
C6—C1—C10	105.39 (14)	C18—C17—C16	119.71 (19)
C5—C6—C1	121.35 (17)	C18—C17—H17	120.1
C5—C6—C7	133.14 (18)	C16—C17—H17	120.1
C1—C6—C7	105.34 (14)	C17—C16—C15	120.49 (19)
O1—C7—C14	111.76 (12)	C17—C16—H16	119.8
O1—C7—C6	100.77 (12)	C15—C16—H16	119.8
C14—C7—C6	117.27 (14)	C16—C15—C14	120.2 (2)
O1—C7—C8	101.26 (12)	C16—C15—H15	119.9
C14—C7—C8	118.27 (14)	C14—C15—H15	119.9
C6—C7—C8	105.00 (13)	C7—O1—C10	98.30 (11)
C13—C8—C9	105.95 (14)	O2—C11—C12	125.1 (2)
C13—C8—C7	114.54 (15)	O2—C11—C9	126.9 (2)
C9—C8—C7	101.66 (13)	C12—C11—C9	107.80 (16)
C13—C8—H8	111.4	O2—C11—H11A	64 (3)
C9—C8—H8	111.4	C12—C11—H11A	115 (3)
C7—C8—H8	111.4	C9—C11—H11A	101 (3)
C11—C9—C8	105.64 (13)	C12—C11—H11B	114 (3)
C11—C9—C10	113.96 (14)	C9—C11—H11B	112 (4)
C8—C9—C10	102.06 (12)	H11A—C11—H11B	107 (2)
C11—C9—H9	111.5	C11—C12—C13	105.70 (18)
C8—C9—H9	111.5	C11—C12—H12A	110.6
C10—C9—H9	111.5	C13—C12—H12A	110.6
O1—C10—C20	111.98 (13)	C11—C12—H12B	110.6
O1—C10—C1	100.73 (12)	C13—C12—H12B	110.6
C20—C10—C1	117.50 (14)	H12A—C12—H12B	108.7
O1—C10—C9	100.68 (12)	O2'—C13—C12	129.4 (2)
C20—C10—C9	118.19 (14)	O2'—C13—C8	123.2 (3)
C1—C10—C9	105.14 (13)	C12—C13—C8	107.28 (16)
C25—C20—C21	118.84 (18)	O2'—C13—H13A	51 (2)
C25—C20—C10	121.40 (16)	C12—C13—H13A	112 (3)
C21—C20—C10	119.73 (17)	C8—C13—H13A	114 (3)
C20—C25—C24	120.7 (2)	O2'—C13—H13B	61 (2)
C20—C25—H25	119.7	C12—C13—H13B	99 (3)
C24—C25—H25	119.7	C8—C13—H13B	112 (2)
C23—C24—C25	120.2 (2)	H13A—C13—H13B	111 (2)
C23—C24—H24	119.9	C11—O2—H11A	49.1 (11)
C25—C24—H24	119.9	C11—O2—H11B	54.1 (9)
C24—C23—C22	120.0 (2)	H11A—O2—H11B	102.4 (16)
C24—C23—H23	120.0	C13—O2'—H13A	52.4 (9)
C22—C23—H23	120.0	C13—O2'—H13B	49.4 (9)
C23—C22—C21	120.1 (2)	H13A—O2'—H13B	100.6 (14)
			
C6—C5—C4—C3	0.4 (3)	C1—C10—C20—C21	−78.9 (2)
C5—C4—C3—C2	0.4 (4)	C9—C10—C20—C21	48.9 (2)
C4—C3—C2—C1	−0.6 (3)	C21—C20—C25—C24	−0.4 (3)
C3—C2—C1—C6	−0.1 (3)	C10—C20—C25—C24	−178.60 (18)
C3—C2—C1—C10	175.47 (18)	C20—C25—C24—C23	−0.6 (3)
C4—C5—C6—C1	−1.0 (3)	C25—C24—C23—C22	0.9 (4)
C4—C5—C6—C7	−175.42 (18)	C24—C23—C22—C21	−0.1 (4)
C2—C1—C6—C5	0.9 (3)	C23—C22—C21—C20	−1.0 (3)
C10—C1—C6—C5	−175.74 (15)	C25—C20—C21—C22	1.2 (3)
C2—C1—C6—C7	176.63 (15)	C10—C20—C21—C22	179.41 (18)
C10—C1—C6—C7	0.02 (16)	O1—C7—C14—C19	15.4 (2)
C5—C6—C7—O1	−153.23 (18)	C6—C7—C14—C19	−100.16 (19)
C1—C6—C7—O1	31.73 (15)	C8—C7—C14—C19	132.40 (16)
C5—C6—C7—C14	−31.7 (3)	O1—C7—C14—C15	−166.71 (14)
C1—C6—C7—C14	153.24 (14)	C6—C7—C14—C15	77.7 (2)
C5—C6—C7—C8	101.9 (2)	C8—C7—C14—C15	−49.7 (2)
C1—C6—C7—C8	−73.15 (16)	C15—C14—C19—C18	0.3 (3)
O1—C7—C8—C13	79.95 (17)	C7—C14—C19—C18	178.16 (16)
C14—C7—C8—C13	−42.5 (2)	C14—C19—C18—C17	0.0 (3)
C6—C7—C8—C13	−175.54 (15)	C19—C18—C17—C16	−0.1 (3)
O1—C7—C8—C9	−33.79 (14)	C18—C17—C16—C15	−0.1 (3)
C14—C7—C8—C9	−156.22 (13)	C17—C16—C15—C14	0.4 (3)
C6—C7—C8—C9	70.72 (15)	C19—C14—C15—C16	−0.5 (3)
C13—C8—C9—C11	−1.02 (18)	C7—C14—C15—C16	−178.37 (16)
C7—C8—C9—C11	118.98 (14)	C14—C7—O1—C10	−176.13 (12)
C13—C8—C9—C10	−120.44 (15)	C6—C7—O1—C10	−50.81 (14)
C7—C8—C9—C10	−0.44 (14)	C8—C7—O1—C10	57.03 (13)
C2—C1—C10—O1	152.25 (18)	C20—C10—O1—C7	176.51 (12)
C6—C1—C10—O1	−31.71 (16)	C1—C10—O1—C7	50.82 (14)
C2—C1—C10—C20	30.4 (3)	C9—C10—O1—C7	−57.02 (13)
C6—C1—C10—C20	−153.59 (14)	C8—C9—C11—O2	169.8 (3)
C2—C1—C10—C9	−103.5 (2)	C10—C9—C11—O2	−79.0 (3)
C6—C1—C10—C9	72.57 (15)	C8—C9—C11—C12	−15.9 (2)
C11—C9—C10—O1	−78.96 (16)	C10—C9—C11—C12	95.3 (2)
C8—C9—C10—O1	34.42 (14)	O2—C11—C12—C13	−158.7 (3)
C11—C9—C10—C20	43.3 (2)	C9—C11—C12—C13	26.8 (2)
C8—C9—C10—C20	156.64 (14)	C11—C12—C13—O2'	148.9 (4)
C11—C9—C10—C1	176.73 (14)	C11—C12—C13—C8	−27.4 (2)
C8—C9—C10—C1	−69.90 (15)	C9—C8—C13—O2'	−159.2 (3)
O1—C10—C20—C25	−16.6 (2)	C7—C8—C13—O2'	89.6 (4)
C1—C10—C20—C25	99.27 (19)	C9—C8—C13—C12	17.4 (2)
C9—C10—C20—C25	−132.91 (17)	C7—C8—C13—C12	−93.8 (2)
O1—C10—C20—C21	165.24 (15)		

### (II) 1,8-Diphenyl-14-oxatetracyclo[6.5.1.02,7.09,13] tetradeca-2,4,6-trien-10-one Hydrogen-bond geometry (Å, º)

Cg1 is the centroid of the C14–C19 ring.

**Table:**

*D*—H···*A*	*D*—H	H···*A*	*D*···*A*	*D*—H···*A*
C5—H5···O2^i^	0.93	2.65	3.472 (3)	147
C12—H12*B*···*Cg*1^ii^	0.97	2.88	3.783 (3)	156

Symmetry codes: (i) *x*−1/2, −*y*+1/2, *z*−1/2; (ii) −*x*+1, −*y*, −*z*.
